# Eighth Annual Meeting of the American Medical Association

**Published:** 1855-06

**Authors:** 


*Eighth Annual Meeting of the American Medical Association.*—This body met on Tuesday, May 1, at Musical Fund Hall, in Philadelphia. During Monday a large number of delegates arrived, and there was a full attendance at the hall on Tuesday, A. M. The hall had been fitted up for the convention. In the lower room of the building was placed a finely executed stone intended for the Washington monument. The stone bears the following inscription:

INSTITUTED

MDCCCXLVII.

Vincit Amor Patriæ.

On the face of the stone was cut, in alto relievo, a most beautiful representation of Hippocrates refusing a bribe tendered him by Artaxerxes, the King of Persia. The stone is four feet by two, and twenty inches in thickness.

The hall is divided into two portions, one for the convention, and the other for the persons who have tickets of admission to witness the proceedings.

At an early hour, a large number of the delegates presented their credentials in the lower room, and their names were duly registered. A committee continued to sit there for the reception of delegates from abroad.

The following officers of the association appeared in their seats:

*President*—Dr. Chas. A. Pope, of St. Louis.

*Secretaries*—Dr. Francis West, of Philadelphia, and Dr. E. S. Lemoine, of St. Louis.

The association was not called to order until half past eleven o'clock.

The President invited all ex-Presidents and ex-Vice-presidents to take seats on the platform.

The first business in order was the report of the committee of arrangement.

Dr. Isaac Hays, of Philadelphia, chairman of the committee of arrangements, said that on behalf of the profession of Philadelphia, he would extend a cordial greeting to the members of the association. It gave them the highest pleasure to have the association among them. The committee had made the best arrangements in their power, to render the sojourn of the delegates agreeable. While this was felt as a duty, it was also a gratification. Eight years have elapsed since this association was first organized in Philadelphia. In every city in which the annual meetings of the society have been held, our delegates have been treated with an elegant hospitality which we cannot pretend to rival. But it was hoped the efforts of the committee would render agreeable the stay of the assembled wisdom of the noblest of professions. Dr. Hays then stated that 337 delegates had registered their names. By general consent the usual calling over of this long list of names would be dispensed with, and the association proceed to the next order of business, which was the calling of the roll.

Dr. West then called the roll.

The address of the President, Dr. Charles A. Pope, of Missouri, was read after the calling of the roll. We give it in full.

Gentlemen—With feelings of grateful pleasure, I meet you, and greet you, on this occasion.

For high and useful purposes have we assembled from the wide extent of our beloved country. The elevation of a noble profession—the promotion of science—the good of humanity—these have been, are, and will continue to be the objects of our association. Whether we have, thus far, done much or little, our sole aim has been the advancement of the best interests of our fellow men. I shall not assert that we have done as much as we might have done, or that the course hitherto pursued by us is so perfect as to admit of no improvement. Were such the fact, and were the association a firmly established institution, I might have experienced more hesitation in the selection of a theme for the present occasion. And since we cannot, as yet, I think, urge such a claim, the few suggestions which I shall offer, are made with becoming diffidence, but at the same time with a deep sense of their importance to the welfare and perpetuity of our association.

Some strictures on our proceedings, in medical and other journals, have appeared within the last year, as well as in previous years. I shall not here blame the authors of them. They are, doubtless, as proud of our noble profession as we, and equally with us, anxious for the advancement of its interests and its honor. I thank them for their suggestions. All of us are ready to hear them and to profit by them. If any more effectual mode of arriving at truth can be devised than that which we have heretofore pursued, all of us are ready to follow it, and would rather thank than quarrel with those who propose it.

Physicians have an almost superhuman mission to fulfil. The goal of their ambition, and their hopes, and their duty, stands at the *ultima thule* of human capacity—nay, rather beyond it. It cannot, indeed, be said that their duties are beyond their powers, but their ambition, their hopes, their wishes certainly are. They would gladly know, not only all the secrets of organization, but those also of Pathology and Therapeutics. To arrive at such knowledge is, perhaps, beyond the attainment of the human mind. Multiform are the elements which enter into the problem of health and disease. Health is, itself, a constant change of composition—diseases are ever varying changes, supervening on this.

Do we know, with all our advancement, and after all the toil of our predecessors for two thousand years, the exact changes in which any disease, the fevers, for instance, consists? And even when we shall have learned these, so as to understand them as well as the most ordinary chemical changes, the ever-varying character of most diseases, and the inward disturbing influences upon them of the mental and moral emotions, would require to follow them, a continued stretch and power of intellect, of which it is doubtful if man be capable. This exactness of knowledge is not, I grant, necessary to the very successful practice of medicine. Our profession can render great and important services to man without it, but with it, it would be still more serviceable. To it our ambition tends. To this perfect knowledge we aspire. Although we may never reach, we can yet eternally approach it. In the vast region of our researches, there is no probability that human genius will ever, Alexander-like, weep for the want of unconquered provinces. Beyond the conquests of the future heroes of the profession, there will always be a boundless field for the ambitious and philanthropic explorer. In the language of a western student, "the science of Medicine, like the liver of Prometheus, is sufficient to glut the eagles of all time."

The object of this association is to do something to advance the profession toward the far-distant goal of perfection—to aid the solution of some of the problems and enigmas of life and organization—to add some material to the growing temple, whose foundations were so firmly laid by the Coan sage, and to do its part, as best it may, in the cause of humanity. Nor do I think that, so far, it has altogether failed. Many valuable contributions to science have been elicited—professional ambition has been stimulated—an *esprit-du-corps* has been successfully evoked and established. The strength of the profession has acquired additional power by the union of its members. This association has been to physicians what the railroad and electric wires are to commerce, and the interchange of useful knowledge to states and nations. It has made us one, and, as I have just remarked, in unity there is power.

This association has stimulated thought. Chaotic and void would forever remain the masses of facts, accumulated by the observations of ages, but for the coördinating and logical power of reason. It sits in judgment on the silent phenomena, as a "refiner of fire, and a purifier of silver." It forces the voiceless facts to mount the tripod of the oracle, and speak forth words of wisdom. The scalpel, the crucible, the microscope, may be subsidiary to its purposes and ends, but they cannot supply its place. Fixed and patient thought, in medicine, as in the other departments of science, is the Aladdin's lamp that lights the footsteps of the discoverer. To stimulate attention and thought, is to accelerate many a new discovery—to hasten the advent and establishment of important principles yet in the womb of the future. May not our association do this more effectually than it has hitherto done?

Let all the contributions be read and attentively considered. Such a course would certainly be more encouraging, as well as more respectful, to their authors. Let the reports be deliberately and fully discussed, and let them go forth to the world with the sanction of the criticisms of the association. This would require time, it is true, but if we have time to meet at all, surely a few days would make but little difference. The good that would be effected would yield a ten-fold compensation for the time employed. Every one must admit that three or four days is too short a time for the association readily to fulfill its annual mission.

I would, moreover, respectfully suggest, that time be taken for the discussion of some of the leading topics of medical philosophy. Among these, may be mentioned the nature, causes and treatment of cholera, yellow fever, et cetera—Hygiene, and the laws of health affecting masses of men—Quarantine—the causes of mortality among children—the chemical and vital doctrines of life. Questions like these, indicated a year in advance for discussion, would excite a carefulness of investigation, and a degree of attention and thought which could not fail to clear away much of the darkness and doubt in which they are yet shrouded. Nothing so sharpens the intellectual powers as public debate. It fixes attention, and strains to the utmost every faculty. I have no hesitation in saying that facts enough have been accumulated to establish great and general principles, of which the medical world is yet in ignorance or doubt. Nothing would contribute more to demonstrate these principles than the collision of matured intellects in public debate. What a mass of facts, and arguments, and demonstration, would be brought to bear, on any of the subjects alluded to, if some of the best minds in the profession were to debate them, after a year's preparation! Observed facts are the crude materials of science—the intellect is the master builder of its august temple.

I make these considerations for your consideration. All the scientific meetings in this country and in Europe, employ more time than ours has hitherto employed. Evidently we must protract our sessions, if we would render them as serviceable to science as they may be. No member of the association will be required to remain longer than suits his wishes or convenience. Some fifty or sixty, more or less, would always be found to listen with eagerness to scientific papers, and engage with pleasure in scientific discussions.

The time has probably arrived for a change in our plan of organization, which will admit of the selection of a permanent place for the future meetings of the association. There are evident advantages incident to both the migratory and stationary plans. These might, perhaps, be easily reconciled and secured. A proposition, if I mistake not, was made some years ago, by the Smithsonian Institution, and I would respectfully suggest, whether it would not be in accordance with the best interests of the association, to hold biennial meetings in Washington, and the alternate ones, as now, at different points of our common country. We might thus secure all the advantages of a fixed abode, in the way of preserving the archives, making collections, etc., whilst by meeting in various localities, we could not fail to excite that wide-spread interest among the profession, and obtain such accessions of new members as would greatly enhance the high and useful objects of our association. Should this proposal meet with your approbation, I would further intimate, that policy would, perhaps, require the meetings of the association at the National Capital, to be held in the years of the short sessions of Congress.

I shall say but little of the legislative duties of the association. I shall say nothing of the propriety or impropriety of getting laws passed to regulate the practice of medicine, and furnish standards for candidates for the doctorate. Perhaps the association can do but little in this respect. Ours is a popular government, and the people are disposed to allow the largest freedom in everything pertaining to medicine, medical schools and physicians. Laws passed against quackery one year are revoked the next. Our country is the paradise of quacks. All good things have their attendant evils, and this unbridled liberty is one of the evils of a popular government. May we not hope, however, that even this evil may disappear, as general education and the cultivation of the masses advance? At any rate, the people are not yet disposed to put down the quacks, nor to require too high a degree of qualification for those of the regular profession. After all, laws can make only mediocre physicians. They can require the candidates to know only so much—to be qualified to a certain degree; and this degree will always be far lower than that to which the true lovers of knowledge would attain, without any legislation on the subject. The greater lights of the profession cannot be manufactured after any process of legislative enactment. Thirst of knowledge, self-love, philanthropy, burning ambition—these make the great physician and surgeon. These have made all the worthies of the past—not legislation. Legislation cannot drive the drone to the proud heights of professional eminence. When these heights are reached, it will be seen that the successful aspirant has been stimulated by a stronger power.

To him the laurel-blossoms of renown and the life-giving mission of his art, are dearer and more attractive than was the mystic bough of the sibyl to the eager Æneasor; than the golden apples, guarded by sleepless dragons, to the Hesperian daughters.

Whatever course you may think proper to pursue, I am sure that your objects will be, the advancement of science—the good of mankind—the honor and glory of the profession. We have the dignity and character of a noble calling to sustain—of a profession which has numbered, for two thousand years or more, some of the wisest and best of men in all countries and all times. It is no trivial matter, to sustain the rank and respectability of a vocation which can boast of a Hippocrates—a Harvey—a Hunter—of the most erudite and beneficent of sages and philanthropists the world ever saw—of a profession which has furnished to every nation its *clarum et venerabile nomen.*

On the eve of the battle of the pyramids, Napoleon exclaimed—"Soldiers! from the height of yon monuments, forty centuries look down upon you." Gentlemen, from the heights of past ages, countless worthies of our God-like profession point and beckon to a goal more elevated than that which attracts legislators and conquerors, Solons and Cæsars.

On motion of Dr. J. Biddle, the thanks of the association were tendered to the President, Dr. Pope, for the able and eloquent address just delivered.

Dr. Hays then announced the various excursions planned by the committee of arrangements for the pleasure of the association.

Dr. Condie introduced a resolution on the subject of permanent membership, which was discussed, and an amendment offered by Dr. Watson, of New York. The whole subject was, on motion of Dr. White, of Buffalo, referred to a committee of three. The chairman appointed Drs. White, of Buffalo, Watson, of New Yoık, and Condie, of Philadelphia, such committee.

A recess was then held, during which each state appointed a member of the nominating committee. Some amusement was created by the delegation from New York. A large number of its members had gathered upon one side of the hall, while a smaller party were in session in the centre of the room, composed of some few of the older members of the profession. "Young America" yielded, and went over to the gray heads just in time to confirm their action, which had been duly arranged beforehand. This piece of generalship was received with a good-natured acquiescence.

On the resumption of business, invitations from Detroit, Nashville, and Chicago, were presented, asking the association to meet in these localities next year.

Dr. D. D. Thompson, of Kentucky, moved that the regular order of business be dispensed with, to take up the amendments to the Constitution offered at the last annual meeting.

The association seemed to have had its dose of constitution, and manifested decided objections to any further tinkering with the organic law. This was a great blow to some of the "prominent members," as it deprived them of their annual delivery of long speeches on a subject which is evidently very interesting to them, if not to others. We felt aggrieved, also, inasmuch as we intended to have had full and accurate reports of these abortive speeches, and to have based on them certain well digested views of our own on the feasibility of pleasing every one.

A report was received through Dr. La Roche, from the committee on prizes. The report stated that the committee had received six essays in competition for the prize offered by the association. But, although these essays evinced much ability and extensive learning, but one was decided to possess those qualities which deserved the award of the prize. The essay was entitled "Statistics of Placenta Prævia." The name of the author was announced as Dr. James D. Trask, of White Plains, New York. (Applause.)

On motion, the report was accepted and the prize essay was referred to the committee on publication.

The committee on epidemics of Missouri, Iowa, Illinois, and Wisconsin, submitted a voluminous report, the abstract of which occupied a long time in reading.

On motion, the report was referred to the committee on publication.

Dr. White, from the committee to whom was referred the resolutions in regard to the permanent members, submitted a report, recommending the adoption of the following resolution:

*Resolved*—That no permanent member who is not present at an annual meeting of this association, shall be required to pay the usual assessment; but no such permanent member shall be entitled to receive a copy of the printed proceedings of the meeting, unless by paying a sum equal to that assessed upon those who were present at such meeting.

The report of the committee was accepted and the resolution was adopted.

Dr. Sanford B. Hunt, of Buffalo, N. Y., from the committee on the hygrometrical state of the atmosphere in various localities, and its influence on health, submitted a report, pending the reading of which the hour of adjournment arrived, and the association adjourned to meet Wednesday morning at nine o'clock.

After the adjournment a large number of the members visited the Pennsylvania Hospital for the Insane.

SECOND DAY.

The association reässembled on Wednesday morning at 9 o'clock.

Dr. J. Atlee, of Lancaster, addressed the convention on the subject of the stone made for the Washington National Monument, by J. A. Beck, a skillful American artist. Dr. A. stated that at a meeting of the association held in Richmond, Va., it was ordered that a stone should be prepared for the monument. Dr. Pearson, of Salem, Mass., who advised that the representation of Hippocrates refusing presents, should be placed on the stone, but before he could consummate the plan the fearful accident happened at Norwalk, which swept him from their midst. It is said that Hippocrates, upon being sent for by Artaxerses, King of Persia, to be the Court Physician, returned as an answer, "tell your master I am rich enough; honor will not permit me to succor the enemies of Greece."

A resolution was adopted that no member be allowed to speak unless his name and residence be announced.

The committee on nominations, reported the following nominations:

*President*—George B. Wood, Penn.

*Vice-presidents*—William M. ——, Ala.; Daniel Tilden, Ohio; D. Humphrey Storer, Mass.; Grafton Tyler, D. C.

*Secretaries*—Francis West, Penn.; R. C. Foster, Tenn.

*Treasurer*—Casper Wistar, Penn.

*Committee on Publication*—Francis G. Smith, Penn.

The President elect, on taking the chair, said that he felt deeply sensible of the honor conferred upon him, and though he had all his life devoted himself to the cause in which we are all engaged, yet he is unaccustomed to preside, and any errors which he may make, he hoped the association would excuse. He assured the convention that he would do his best to justify his appointment as presiding officer.

The committee further reported on the subject of the next place of meeting, and recommended Nashville, Tennessee.

A motion was made to substitute District of Columbia, in place of Nashville. Laid on the table.

Dr. A. B. Palmer, of Michigan, moved to substitute Detroit, Michigan, in place of Nashville.

A resolution was offered that the whole subject be referred to a special committee of five, to be appointed by the chair. Laid on the table.

The question was now taken, on the motion of Dr. Palmer, and it was adopted. Detroit, therefore, is the next place of meeting. The result was unanimously approved by rounds of applause.

Dr. R. C. Foster, appointed secretary, tendered his resignation, which was accepted.

Dr. Brodie, of Michigan, was appointed to fill the vacancy.

Dr. Sanford B. Hunt resumed the reading of his report on the hygrometrical state of the atmosphere, in various localities. Pending the reading of the account, a motion was made and adopted, "that the desultory conversation of members be dispensed with." The report abounds with interesting facts on the subject of epidemical diseases, during the summer of 1854.

It was accepted, and referred to the committee on publication.

A resolution was adopted, providing that a committee be appointed to confer with the directors of the several railroad and steamboat companies, with the view of having commutation tickets issued to the delegates to the convention, to be held at Detroit next year.

Dr. Frank H. Hamilton, of Buffalo, N. Y., then submitted a report on "Deformities after Fractures." The report was not complete, the author having only considered fractures of the ossa nasi, septum nasi, superior maxilla, inferior maxilla and clavicle. Copious statistics accompanied each fracture.

Dr. Hamilton said he had a word to say which did not belong to the report. Prosecutions for malpractice have become so frequent that surgeons were alarmed, and not a few were abandoning the profession, or refusing altogether to undertake the treatment of grave surgical accidents, and especially of fractures. So frequent were these prosecutions that members were no longer surprised at such statements. If they had heard the speaker say that lawyers were abandoning their profession from this cause, they would have been startled, but to us the fact is familiar.

It is proper for us, then, to interrogate ourselves. Why is it that we are held to an accountability so much more strict than any other professional men, or than any other artizans? Is it because there are jealous and designing men in our own ranks who instigate these suits? No doubt such men may be found, but only as an exception. The fact is that surgeons have sometimes been mulcted in damages simply because the jury believed, from the united character of the medical testimony, that it was a conspiracy, and the more conclusive the testimony, the more certain, with some jurors, is the defendant to suffer.

Is it chargeable to the members of another profession—to the lawyers? There may be some men in the profession of law, also, who, driven by the sheer necessity of their circumstances—by their extreme poverty, or who, without any such apology, with only loose notions of right and wrong, encourage and undertake such suits—such are the men who hang about the tombs in New York, and who may be found, more or less, in every town—but the speaker has reason to believe that honorable and intelligent lawyers seldom countenance these prosecutions. That eminent jurist of the state of New York, Joshua Spencer, has told Dr. Hamilton, that for himself he does not think he ever commenced a suit of this character, although he has been frequently retained as counsel, and he believes his brethren, generally, look upon these complaints with suspicion and refuse to meddle with them.

Where, then, must we look for an answer to the question, Why are these prosecutions against surgeons so frequent? Let the gentlemen be assured, the causes are to be found in the *very imperfections of our art, and in our own unwillingness to admit these imperfections.* Surgeons have claimed too much, and it cannot certainly be expected that the world will demand of them less than they claim for themselves. Again and again surgeons have said that a fracture of the femur might be generally made to unite without any shortening, while the fact is not so. Malgaigne, who is eminently an honest man, says, to make this bone unite in an adult person, where the fracture is sufficiently oblique to prevent the ends from supporting each other, is "simply impossible" *(simplement impossible.)*

Let the profession be wiser in future and acknowledge that they cannot perform impossibilities.

Dr. Charles Hooker, of New Haven, read a report upon the "Diet for the Sick." The document lays down certain laws for the government of diet in the various diseases "flesh is heir to," and specific articles that may be given to patients. Referred to the committee on publication.

A resolution expressing the thanks of the association to the ex-President and other officers, for the ability with which they have discharged their duties, was adopted.

At this period the association adjourned to proceed to Independence Hall. On arriving at this place, the delegates were introduced to Mayor Conrad, by Dr. Isaac Hays, chairman of the committee of arrangements.

Dr. Hays briefly addressed his Honor the Mayor, giving an outline of the history of the association, and alluding to the patriotism of the profession as exemplified in Rush, Warren, and others, closed as follows:

But I may assure you that the spirit which animated our ancestors, if it appear dormant, is not extinct in our bosoms, and that while standing on this spot—the shrine sacred to Human Liberty,—we experience feelings akin to those which the inspired Law-giver must have felt when he heard the voice calling to him out of the midst of the burning bush—"Put off thy shoes from off thy feet, for the place whereon thou standest is holy ground." (Loud Applause.)

The truly eloquent reply of Mayor Conrad will well repay perusal.

### REPLY OF MAYOR CONRAD.

Mr. Chairman of the Committee of Arrangement, I thank you in the name of the community which I have the honor to represent, for your eloquent introduction of our friends to the authorities of the city, and to this, the Hall of Independence.

Gentlemen of the American Medical Association, I am proud of the privilege of extending to you, in the name of the government, and of the people, of Philadelphia, a most cordial welcome. (Applause.)

I bid you welcome to our city—a city which, deriving a cherished distinction from the profession which you adorn, is eager, now and ever, to requite it in her tribute of respect for its professors. I welcome you to our people, whose intercourse, for many a year, with you or your brethren, has inspired a feeling which, reserved as we are sometimes said to be, will, doubt not, burst into earnest and unambiguous expression, before you leave us. (Applause.)

I welcome you, gentlemen, to this Hall, but not as strangers, or the sons of strangers—for it is your own. As the temple ard territory of Delphos, in the wildest domestic perturbations of Greece, afforded one sacred area over which the cloud of discord never gathered, one altar whose worship was never invaded, this spot, consecrated to our common American glory, knows no lines of latitude, and belongs, in truth, no more to us, whose peculiar privilege it is to inherit its guardianship, than to our brothers—*to you.* In coming hither, therefore, you *come home.* (Applause.) These precincts have been hallowed, for all time, by the heroic virtues of your and our fathers. This is the fountain from the which the living waters of American liberty were first drawn, and it is, therefore, most sacred—(woe to the generation in which it ceases to be sacred!)—but, like the well of the Patriarch, all the tribes of Liberty's Israel own here an equal right, and owe here an equal homage. (Great applause.)

In no sense, then, can I greet you as strangers, for yours are names familiar to every American proud of the science of his country, and those who are united, by this association, in a cause so lofty as that eloquently characterized by your chairman, may not only claim the universal and acknowledged privileges of the republic of minds, but the rights of a nearer and dearer charter, the brotherhood of beneficence—the kindred claims of noble hearts, knit in the highest and holiest of human aspirations. In this spirit, with the most fervent and fraternal sentiments of respect and regard, I greet and welcome you.

You are right, Mr. Chairman, in claiming, amid the associations which hallow these precincts, a peculiar privilege for your profession—a profession which not only sprinkled, with the first blood of the revolution, the highest altar upon which valor vowed and dedicated our country to freedom—I refer, as you have referred, to Dr. Warren and Bunker Hill—but which, in every struggle for the enlargement and enlightenment of human destinies, has been eminently distinguished for courage, zeal and fidelity to the rights of man. You have, therefore, a peculiar right to claim kindred here, and have that claim allowed; and within these walls which witnessed the zeal of Rush, it would be treason to virtue to forget that one of the lights of your profession shed glory upon the solemn debates of this hall, and was foremost among those that bade yonder bell, (preserved and devoted to the veneration of posterity,) with its iron tongue to "*proclaim liberty throughout all the land to all the inhabitants thereof.*" (Continued applause.)

It is the glorious peculiarity of your profession that, while ambition, in its ordinary and most applauded paths, plays the part of *the Destroyer*, and wins glory at the expense of human life and happiness, you and yours, with a more exalted civilization and a nobler heroism, have ever *sought to save.* Next to the highest of all human courage—if, indeed, it be merely *human*—that of the martyrs of religious truth, the courage of the physician, whether on the battle-field or in the lazar-house, the courage of science and humanity, is the most sublime, and best entitled to the *clarum et venerabile nomen.* The vulgar courage of the warrior, under the base stimulus of passion or the low greed of applause, can hardly be compared to the noble intrepidity of the surgeon, who gleans, in the ruthless and red-handed reaper's path, the leavings of battle; and still less with the hero of the hospital, who encounters the grim antagonists in the horrid silence and gloom of the pestilence. Imagination can hardly embody an instance of human courage and virtue more sublime and unearthly than that of the physician, who in the midnight of a plague-stricken city, midst the fœtid solitudes of its alleys, and entering the devoted hovel of the wretched, ministers—while only pestilence, and misery, and death, and God look on, to the perishing. I need not step from this spot to grasp the hand of many a hero who claims no laurel—many a noble philanthropist whose sacred labors in scenes like these, have been unmarked, save by the eye that never slumbers, and remembered only by Him, who alone can reward.

To such a profession, one venerable from its antiquity, noble from the grandeur of its objects, illustrious from its achievements, and which demands every aid and energy of genius and science, of head and heart that dignifies the race, it is not strange that, go where it may, a ready homage greets and a ready blessing attends it. In our own city, all that is noble in patriotism, all that is exalted in science, all that is bright and beautiful in the arts that refine society, all that is lovely and cherished and holy in private life, combine to render the profession sacred and dear to us.

There are few living to whom some one death in the past is not the sole event and solitary memory of the survivor's life—to him a lonely pyramid in the melancholy desert; and to such a mind and memory, the debt of the death-bed, where science, rendered holy by its office, ministered, though never paid is never repudiated. I never knew a good man, still less a good woman, who had not such a debt—a debt which bankrupt gratitude cherished with its holiest affections and devoutest memories.

In these times, when the omnipotence of associated effort is invoked for so much that is of dubious merit, it is a gratifying spectacle to behold the enlightened professors of the most exalted of all arts—men sage and grave, unselfish and unaspiring, forsaking the homes to which they are bound by the affections and the afflictions of thousands, by wealth and fame and influence, to wander wearily away upon a pilgrimage of hundreds of leagues, in the cause and interests of the human family, its security, its health and happiness. For more than ten years, the representatives of your profession have gathered in convention. What other body of our citizens have made a like effort—a like sacrifice? Selected from the most eminent of the profession, the delegates have been men whose years, like their virtues, were many. How difficult must have been to them the effort to burst through the bonds of a relying and clinging practice! How great the labor and how heavy the sacrifice! They have already visited in this duty, the cities of every section of our wide country. How many have fallen by the wayside? How many martyrs could you not thus number in this cause? How many of the good and great of the profession have, in these benevolent pilgrimages, joined the ranks of the thousands who have sacrificed themselves, at the requisitions of duty, as recognized and enforced by your self-imposed laws—joining the dead in the effort to aid the living. The epitaph of the Spartans at Thermopylæ, might well commemorate the virtues and the fate of these martyrs. But if the cost has been great, the results have been commensurate.

Of the professional advantages attained, though I know them to be invaluable, I will not presume to speak; but I may be permitted to state, as health is the most important subject of municipal provision and care, that the transactions of the association, which I have examined with great interest, comprise much that merits the attention, and will reward the respectful consideration of the municipal government of the Union.

It is natural that Philadelphia should feel, as she does feel, a profound interest in the cause of medical education in this country. She cannot, of course, forget that it was here that the first medical college was established in this country; that its merits and success extorted a reluctant trans-atlantic tribute of admiration, and that progressing rapidly, but wisely, it achieved and maintained an equality with the most celebrated institutions of the old world. As the cause of medical education has expanded, and institutions worthy of the cause and country have sprung up, each triumph, thus attained has been regarded here as the successful outbursting of an offshoot from the primary effort; and Philadelphia, while rejoicing in the expansion and elevation of medical education throughout the land, has almost fancied—so earnest is her interest in medical education—that she had a right to indulge a parental pride in all that advances that interest.

These genial feelings have been maintained, in all their early and fervid freshness, by constant intercourse with all sections of our country. The ingenuous and gallant youths that have come hither for medical instruction have, in their unstudied intercourse, exhibited the character of their respective states in a light so generous and exalted as to win our affections not only for themselves, but for the communities and states which could exult in them as their own. Winter after winter, we have had hundreds of these noble young spirits among us here. And let me remark, that rigorous as I am said to be in the administration of the law, I have yet to know the first occasion to rebuke, much less to punish, a medical student. We have found them as gentle and decorous in their deportment as they are exalted in their aspirations; and had Philadelphia—eminently catholic in her affections for her sister communities—needed a lesson of love and loyalty, these young missionaries would have taught it. This interchange of sympathies has endured for the third of a century, (may it last forever!) Her youths who formerly bore those sentiments to the remote sections of our republic, stand before me now as the revered sages and ornaments of their profession, meeting here the evidences of a reputation which had preceded them, and has long been cherished by us. And who can tell what has been the results of this kindly interchange of kindly feeling? It has doubtless been felt in every community, social, and political relation of life, correcting the prejudices, harmonizing the discords, and subduing the dangers of our common country.

We realize these facts. We recognize in the members of an enlightened profession like yours, so many patriots and philanthropists engaged in the great and general interests of the human race, and, apart from the more scientific acquisitions of your annual meetings, we perceive, in them, results auspicious to all that we cherish, all that is kindly, forbearing and conservative between man and man, party and party, state and state, section and section; and so regarding them we hail and greet you with a welcome as sincere and cordial as the heart can forge or the tongue can utter. (Loud applause.)

On reässembling at the Musical Fund Hall, it was resolved to visit the High School at 12 o'clock on Thursday, (to-day.)

On motion, the thanks of the association were tendered to Mayor Conrad, for the very cordial manner in which he had received the delegates in Independence Hall, and that a copy of his speech be printed in connection with the proceedings of this association.

Dr. Thompson, of Delaware, offered a preamble and resolutions providing for the appointment of a committee from each state ro report on medical topography and epidemics. Ordered to lie over till next day.

Dr. Isaac Wood, the Treasurer, made a report, from which it appears that during the year the sum of \$3216 30½ was received, of which there is a balance remaining amounting to \$1115 26.

Dr. Francis Condie, from the committee on publication, made a report in reference to certain charges made against the committee and Dr. Meigs, which gave rise to considerable discussion.

Dr. Stewart offered a resolution, expressing thanks for the manner in which the committee on publication had performed their arduous labors. Pending the discussion, the resolution and the report were both withdrawn.

Dr. Watson offered a resolution appropriating the sum of $1000 to defray the expenses of the committee to have prepared the stone for the National Monument. Adopted.

Dr. Mauran called up a preamble and resolution expressive of thanks to those United States Senators who so earnestly and ably advocated the passage of the bill relative to sickness on shipboard, as suggested by this Association.

The resolution was adopted, and copies ordered to be sent to the Senators.

Dr. Francis West, Secretary, read a paper from Dr. William H. Byford, of Evansville, Ind., on the "Pathology and Treatment of Scrofula," which, on motion, was referred to the committee on publication.

Dr. N. S. Davis, of Chicago, Ill., submitted a report on the nutritive qualities of milk, and the influence produced thereon by pregnancy and menstruations, in the human female, and pregnancy in the cow; and also on the question whether there is not some mode by which the primitive constituents of milk can be preserved in their purity and sweetness, and furnished to the inhabitants of cities in such quantities as to supersede the present defective and often unwholesome modes of supply.

The report was referred to the committee on publication.

The hour of adjournment now arrived.

At 4 o'clock yesterday afternoon, the delegates, accompanied by many ladies, visited Girard College.

[Want of room compels us to lay over, until next month, the third and fourth day's proceedings.]

